# Clinical features and genetic spectrum of children with primary ciliary dyskinesia in central China: a referral center retrospective analysis

**DOI:** 10.3389/fphar.2025.1526675

**Published:** 2025-06-13

**Authors:** Xiaoning Zhang, Xiuhong Jin, Zhiying Zhang, Yanqiong Wang, Xiangfeng Zhang, Zhen Dong, Haiming Yang, Yuelin Shen

**Affiliations:** ^1^ Respiratory Department, Children’s Hospital Affiliated to Zhengzhou University, Henan Children’s Hospital, Zhengzhou Children’s Hospital, Zhengzhou, China; ^2^ Respiratory Department II, National Clinical Research Center for Respiratory Diseases, Beijing Children's Hospital, Capital Medical University, National Center for Children's Health, Beijing, China; ^3^ Department of Respiratory Diseases, Pediatric Research Institute of Xinjang Uygur Autonomous Region, Children's Hospital of Xinjang Uygur Autonomous Region, Xinjiang Hospital of Beijing Children's Hospital, The Seventh People's Hospital of Xinjiang Uygur Autonomous Region, Urumqi, China

**Keywords:** primary ciliary dyskinesia, central China, clinical feature, genotype, children

## Abstract

**Background:**

Despite growing awareness of primary ciliary dyskinesia (PCD) in northern China, few cases have been reported in central China, the most populous region in the country. This study aimed to describe the clinical phenotype and genotype of children with PCD in central China.

**Methods:**

We retrospectively recruited 15 patients with PCD from January 2018 to July 2024. The demographic data, clinical characteristics, laboratory and imaging findings were reviewed to clarify the clinical features. Whole exome sequencing was conducted to identify the genotype.

**Results:**

The mean age at diagnosis was 8.2 ± 4.8 years. All 15 patients (100%) experienced recurrent wet cough; 93.3% (14/15) had sinusitis, 80.0% (12/15) had otitis media, and 46.7% (7/15) had neonatal respiratory distress. Chest computed tomography revealed that 93.3% (14/15) had nodular shadows and tree-in-bud signs, and 80.0% (12/15) had varying degrees of bronchiectasis. The most common pathogen in the airway was *Haemophilus influenzae* (9/15, 60.0%). The genes with the highest incidence of variants were *DNAH5* (6/13), followed by *DNAH11* (3/13). The *DNAAF2*, *DNAH9*, *DNAAF6*, and *DNAAF3* genes each were mutated once. Fifteen novel variants were identified.

**Conclusion:**

PCD is underdiagnosed in central China. The phenotype is characterized by a significant male predominance. Additionally, the incidence of neonatal respiratory distress and situs inversus is notably lower compared to Western countries. The most frequently identified gene responsible for PCD was *DNAH5*.

## Introduction

Primary ciliary dyskinesia (PCD), also known as primary ciliary immobility syndrome or immotile cilia syndrome, is a rare hereditary disease caused by defects in cilia structure and cytoplasmic proteins involved in cilia assembly. To date, more than 50 pathogenic genes associated with PCD have been identified. The majority exhibit an autosomal recessive inheritance pattern, whereas two—DNAAF6 and RPGR—follow an X-linked mode of inheritance. Major clinical manifestations include recurrent respiratory infections, bronchiectasis, sinusitis, otitis media, visceral transposition, and infertility. The incidence of PCD varies by ancestry and geographic region. Previous studies have reported an incidence at approximate 1 in 10,000–20,000 individuals worldwide (Niziolek et al., 2022), with a notably higher prevalence in the Asian population. In China, PCD is frequently underdiagnosed or misdiagnosed, likely due to its low prevalence, limited awareness of the disease, and challenges in diagnostic accessibility. A systematic review by Peng B et al., which analyzed data from Embase, PubMed, Web of Science, and Chinese databases from 1981 to 2021, identified only 244 reported cases of Chinese patients diagnosed with PCD (Peng et al., 2022). Central China, which primarily includes Henan, Hubei, and Hunan Provinces, is the most populous region. However, it has reported relatively few cases, all of which are individual case reports (Guo et al., 2017; Jiang et al., 2022; Lu et al., 2021; Liu et al., 2022; Gong et al., 2023). In this study, we retrospectively analyzed the clinical features and genetic spectrum of children with PCD in central China to enhance disease awareness and improve management strategies.

## Methods

### Human subjects

A single-center retrospective analysis was performed on Chinese children with PCD hospitalized in the Respiratory Department of Children’s Hospital Affiliated to Zhengzhou University from 1 January 2018, to 30 July 2024. The diagnostic criteria for PCD were based on the Consensus Guidelines from European Respiratory Society (Lucas et al., 2017), the American Thoracic Society (Shapiro et al., 2018), or both (Guan et al., 2021). PCD was diagnosed based on at least one of the following criteria: recognized ciliary ultrastructural defect; biallelic pathogenic variants in a PCD-associated gene; at least two of the four key clinical features for PCD, combined with low nasal nitric oxide (nNO) level (excluding cystic fibrosis), namely, unexplained neonatal respiratory distress in a term infant, year-round daily cough beginning before 6 months of age, year-round daily nasal congestion beginning before 6months of age, or organ laterality defect; and Kartagener syndrome, which was considered to be PCD even if the previously mentioned criteria were not fulfilled completely.

Clinical data were collected from all enrolled patients, including demographic information, primary symptoms, chest and sinus computed tomography (CT) findings, gastroenterography results, sputum culture analyses, treatments, and outcomes. Monthly follow-ups were conducted after discharge to monitor patient progress. The clinical characteristics of individual patients are presented in Table 1.

**TABLE 1:** Demographic and clinical features of 15 Chinese children (14 families) with PCD who presented to the referral center in central China.

Patient no.	Age of symptom onset, year	Age at Dx, year	Sex	Clinical presentation	nNO, nL/min	Sputum pathogens	Pulmonary function at Dx, %	
							FEV_1_, %	FVC, %
1	0.2	7.0	F	1/3/4/5	11.6	*H. influenzae*	81	81
2	0.2	0.8	M	1/3/4	9.8	*H. influenzae*	ND	
3	0.1	11.0	M	1/2/3/4/5	16.5	*H. influenzae*	85	78
4	0.1	0.2	M	1/2/3/4	22.5	None	ND	
5	3.0	14.0	F	1/3/4/5	7.5	*H. influenzae*	65	83
6	4.0	8.0	M	1/3/4/5	20.3	*H. influenzae*	71	65
7	7.0	16.0	F	1/3/4/5	15.7	*S. pneumoniae/P. aeruginosa*	80	77
8	0.1	11.0	M	1/2/3/4/5	18.5	*H. influenzae*	78	72
9	10.0	13.0	M	1/3/5	16.0	None	80	78
10	0.1	9.0	M	1/2/3/4/5	18.7	None	84	83
11	0.1	0.8	F	1/2	28.5	*P. aeruginosa*	ND	
12	0.2	9.0	M	1/3/4/5	41.8	*H. influenzae*	82	77
13	0.1	5.8	M	1/2/3/4/5/6	19.5	*H. influenzae*	82	74
14	0.1	7.5	M	1/2/3/4/5/6	16.8	*A. baumannii*	80	76
15	2.0	10.0	M	1/3/5	23.8	*H. influenzae*	78	80

Sex: F, female; M, male.

Clinical presentation: 1, Wet cough; 2, Neonatal respiratory distress; 3, Sinusitis; 4, Otitis media; 5, Bronchiectasis; 6, Situs inversus.

Sputum pathogens: *H. influenzae*, *Haemophilus influenzae*; *P. aeruginosa*, *Pseudomonas aeruginosa*; *S. pneumoniae*, *Streptococcus pneumoniae*; *A. baumannii*, *Acinetobacter baumannii*.

Pulmonary function: FEV_1_, forced expiratory volume in the first second; FVC, forced vital capacity.

This study was approved by the Ethics Committee of Children’s Hospital Affiliated to Zhengzhou University (2024‐KY‐0008), and written informed consent was waived.

### Nasal nitric oxide (nNO) measurement

nNO was measured using a Nakulen breath analyzer (Sunvou-CA2122 model) in children over 3 years old. Two hours prior to the test, the child refrained from consuming nitrogen-rich foods and avoided strenuous exercise. For children older than 5 years, NO values were tested using the whistling method. During the procedure, the child held a whistle in their mouth and inserted an olive-shaped nasal plug into their right nostril. Nasal gas was collected while the child continuously whistled in one breath, and the concentration of nNO was measured. For children younger than 5 years, nNO was measured during tidal breathing. The nNO value (nL/min) was calculated by multiplying the nNO concentration (ppb) by the sampling flow rate (0.3 L/min). The results are presented in Table 1.

### Pulmonary function tests

Standard pulmonary function tests were conducted for children aged 6 years and older using a spirometer (MS-IOS, JAEGER). The assessments followed standardized protocols to ensure accuracy and reproducibility. Before the procedure, children received clear instructions and were encouraged to exert maximal effort for reliable measurements. The results for forced expiratory volume in the first second (FEV1) and forced vital capacity (FVC) are presented in Table 1.

### Whole-exome sequencing (WES)

Genomic DNA was extracted using a QIAamp Blood Midi Kit (QIAGEN). The amplified DNA was captured using the GenCap WES capture kit. Whole exons and 20 bp of the flanking intronic regions were sequenced on DNBSEQ (DNBSEQ‐T7) platform with 150 bp paired‐end reads. After sequencing, the clean reads were mapped to the UCSC hg19 human reference genome using BWA software (http://bio-bwa.sourceforge.net/). SNP and InDel variants were detected using the parameter driver of Sentieon software. These variants were further annotated by ANNOVAR software (http://annovar.openbioinformatics.org/en/latest/), and associated with multiple databases, including 1,000 genome, ESP6500, dbSNP, EXAC, HGMD, and predicted by SIFT, PolyPhen‐2, MutationTaster, GERP++. The pathogenicity of variants was assessed according to the guidelines of the American College of Medical Genetics and Genomics. Potential pathogenic variants were verified for probands and their parents through Sanger sequencing using an ABI3730xl sequencer (Applied Biosystems). If WES identifies a large homozygous deletion, quantitative PCR (qPCR) should be performed to verify its parental origin.

## Results

### Demographic and clinical features

A total of 15 pediatric patients (11 males and 4 females) from 14 Chinese families, who presented to the Children’s Hospital affiliated with Zhengzhou University, the main referral center for children with PCD in central China, were recruited for this study. Only Patients 1 and 2 were siblings (Dong et al., 2023), while the remaining cases were sporadic. The age at diagnosis was 8.2 ± 4.8 years, ranging from 0.2 to 16.0 years. The age of symptom onset was 1.8 ± 3.0 years. None of the patients were products of consanguineous unions, nor did they have a family history of PCD. The detailed clinical manifestations and laboratory findings of the patients are shown in Table 1.

All 15 patients (100%) experienced recurrent wet cough. Sinusitis was observed in 93.3% (14/15), otitis media in 80.0% (12/15), and neonatal respiratory distress in 46.7% (7/15). Among them, four patients (Cases 2, 4, 13, and 14) underwent gastroenterography. Situs inversus totalis was identified in two cases (Cases 13 and 14), while the remaining two (Cases 2 and 4) exhibited a tortuously elongated duodenum or colon. Additionally, Case 4’s cranial magnetic resonance imaging (MRI) revealed periventricular nodular heterotopia (PNH), and echocardiography confirmed a patent ductus arteriosus (PDA). Furthermore, Case 6 had an IgG subclass deficiency, whereas Case 11 presented with hydronephrosis.

**FIGURE 1:**
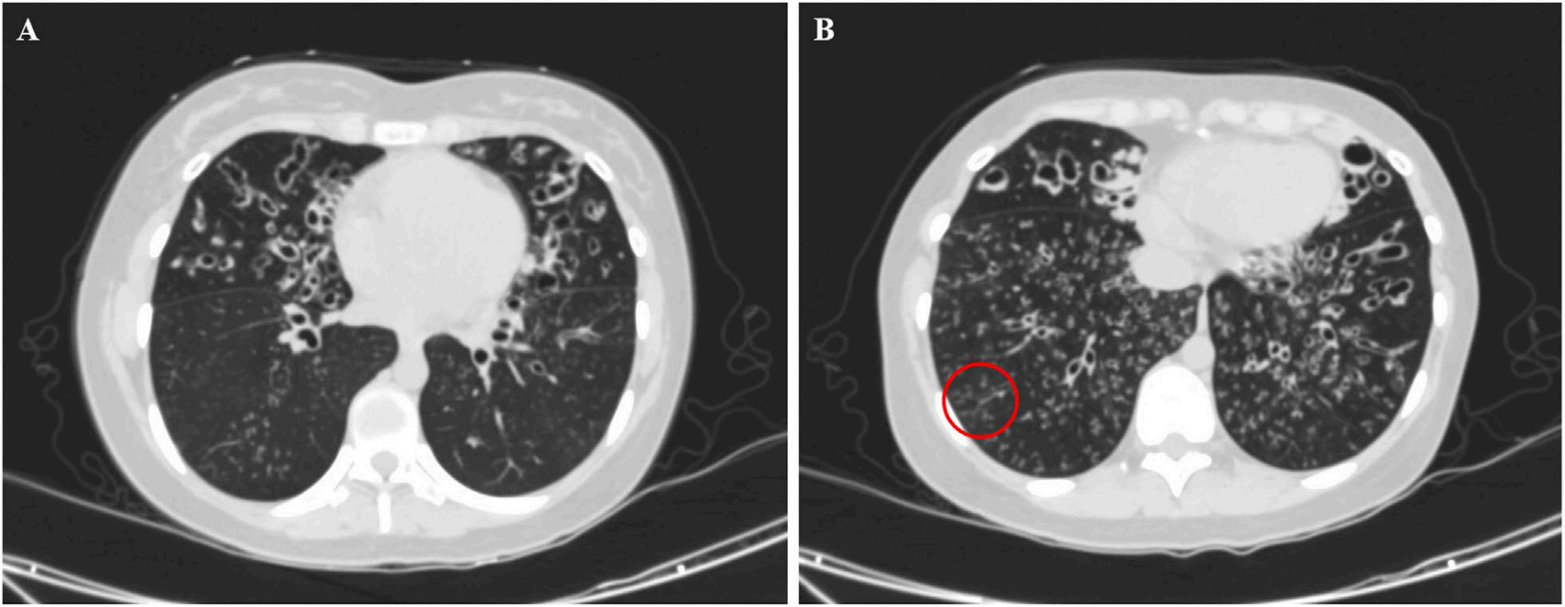
Chest computed tomography image of a 14-year old female patient with PCD on admission (Case 5), showing bronchiectasis in the middle lobe of the right lung and the upper lobe of the left lung **(A)** with nodular shadows and tree-in-bud signs (**B**, red circle).

All patients underwent nNO measurement, and the nNO levels were lower than 77 nL/min. Twelve patients demonstrated obstructive ventilatory dysfunction on pulmonary function tests. All 15 patients underwent chest CT scans. Except for three patients (Cases 2, 4, and 11) who were under 3 years old, the remaining 12 patients exhibited varying degrees of bronchiectasis. The bronchiectasis was predominantly located in the middle and lower lobes of the right lung, each observed in 11 out of 12 patients (91.7%). One patient had undergone lobectomy before admission. Except for Case 4, the remaining 14 patients (93.3%) exhibited nodular shadows and tree-in-bud signs. Four children (Cases 1, 3, 7 and 15) exhibited mucous plugging, while two children (Cases 10 and 13) exhibited atelectasis. The chest CT scans of Case 5 revealed bilateral bronchiectasis, along with nodular shadows and tree-in-bud signs, as shown in Figure 1.

At least two sputum culture specimens were successfully obtained from 15 patients and confirmed to contain a pathogenic species when identical pathogens were detected in at least two separate specimens. The most frequently isolated pathogen was *Haemophilus influenzae* (*H.influenzae*) (9/15, 60.0%), followed by *Pseudomonas aeruginosa* (*P.aeruginosa*) (2/15, 13.3%), *Streptococcus pneumoniae* (*S.pneumoniae*) (1/15, 6.7%), and *Acinetobacter baumannii* (*A. baumannii*) (1/15, 6.7%).

All patients received pulmonary rehabilitation therapy from the time of diagnosis, utilizing postural drainage, chest percussion, and breathing exercises according to their age. Cases 1 and 2 underwent bilateral myringotomy with tube insertion. Case 6 received immunoglobulin infusions of 12.5 *g* every 3 months. All 15 patients attended regular outpatient follow-ups, with their conditions remaining stable without any deterioration or severe complications.

### Genetic testing findings

**FIGURE 2:**
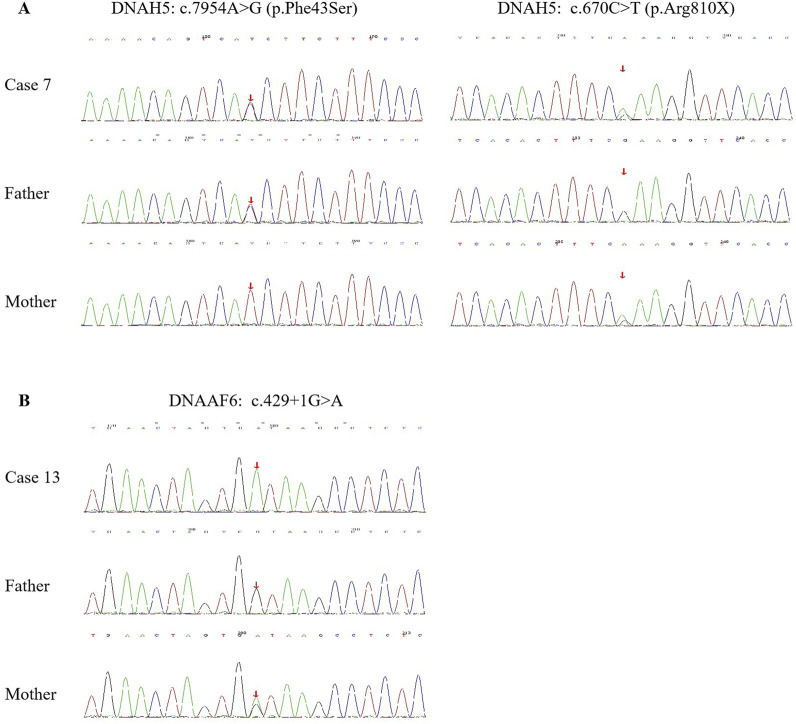
Sanger DNA sequencing results of the *DNAH5* gene in Case 7 **(A)** and the *DNAAF6* gene in Case 13 **(B)** with PCD, as well as the carrier status of their parents.

**FIGURE 3:**
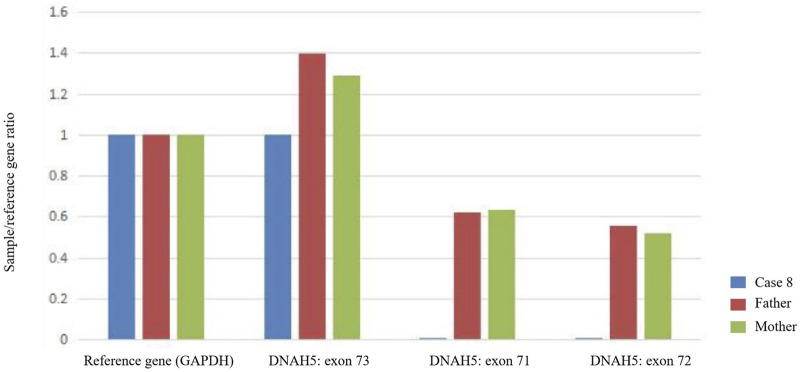
Quantitative PCR (qPCR) demonstrates that Case 8 has a complete loss of copy number for exons 71 and 72 of the *DNAH5* gene (homozygous deletion), whereas the father and mother each carry a single copy (heterozygous deletion).

Fourteen children from 13 families underwent genetic testing, revealing a total of 23 different variants through sequencing, including 15 novel variants. The detailed genetic findings are presented in Table 2. Among these, 92.3% of the families (12/13) exhibited autosomal recessive inheritance (eleven were compound heterozygous, and one was homozygous), with one case showing X-linked recessive inheritance. Specifically, 46.2% had DNAH5 variants (6 families), 23.1% had DNAH11 variants (3 families). DNAAF2, DNAH9, DNAAF6, and DNAAF3 variants were each found in one family. The Sanger DNA sequencing results for the DNAH5 gene in Case 7 and the DNAAF6 gene in Case 13 are presented in Figure 2. The qPCR results confirming the homozygous deletion of the DNAH5 gene in Case 8 are shown in Figure 3.

**TABLE 2:** Genetic features of 14 Chinese children (13 families) with PCD who presented to the referral center in central China.

Patient no.	Gene	Nucleotide change	Amino acid change	dbSNP	Type of variant	Source	Pathogenic significance	Genotype	Inheritance pattern	Reported/novel
1/2	*DNAAF2*	c.156C>A	p.Y52*	rs2139584419	Nonsense	M	Pathogenic	Compound heterozygous	AR	Reported
		c.177dup	p.E60Rfs*3	NA	Frameshift	P	Pathogenic			Reported
3	*DNAH11*	c.2135dup	p.N713Kfs*9	NA	Frameshift	M	Pathogenic	Compound heterozygous	AR	**Novel**
		c.8579G>A	p.G2860D	rs749830026	Missense	P	Uncertain			**Novel**
4	*DNAH5*	c.11178_11181dup	p.L3728Vfs*37	NA	Frameshift	M	Pathogenic	Compound heterozygous	AR	**Novel**
		c.8030G>A	p.R2677Q	rs886043448	Missense	P	Uncertain			Reported
5	*DNAH5*	c.11029-2A>T	-	rs369312501	Splicing	M	Pathogenic	Compound heterozygous	AR	**Novel**
		c.5563dup	p.I1855Nfs*6	rs752925056	Frameshift	P	Pathogenic			Reported
6	*DNAH11*	c.1452T>G	p.F484L	rs755256285	Missense	M	Uncertain	Compound heterozygous	AR	Reported
		c.727A>G	p.I243V	rs189000268	Missense	P	Uncertain			Reported
7	*DHAH5*	c.670C>T	p.R224*	rs771463510	Nonsense	M	Pathogenic	Compound heterozygous	AR	Reported
		c.7954A>G	p.M2652V	NA	Missense	P	Uncertain			**Novel**
8	*DNAH5*	c.12034-?_12499+?del	-	NA	Large deletion	M/P	Pathogenic	Homozygous	AR	**Novel**
9	*DNAH5*	c.4356-158T>G	-	rs1376282258	Sequence variation	M	Uncertain	Compound heterozygous	AR	**Novel**
		c.8839A>G	p.T2947A	NA	Missense	P	Uncertain			**Novel**
10	*DNAH5*	c.8030G>A	p.R2677Q	rs886043448	Missense	M	Uncertain	Compound heterozygous	AR	Reported
		c.12779A>G	p.D4260G	NA	Missense	P	Uncertain			**Novel**
11	*DNAH11*	c.5500C>T	p.R1834C	rs180897552	Missense	M	Uncertain	Compound heterozygous	AR	**Novel**
		c.13183C>T	p.R4395*	rs757784023	Nonsense	P	Pathogenic			Reported
12	*DNAH9*	c.13306C>T	p.P4436S	rs753449749	Missense	M	Uncertain	Compound heterozygous	AR	**Novel**
		c.1670C>T	p.P557L	rs372282496	Missense	P	Uncertain			**Novel**
13	*DNAAF6*	c.429 + 1G>A	-	NA	Splicing	M	Pathogenic	Heterozygous	XLR	**Novel**
15	*DNAAF3*	c.289G>C	p.A97P	NA	Missense	P	Uncertain	Compound heterozygous	AR	**Novel**
		c.1-?_1827+?del	-	NA	Large deletion	M	Pathogenic			**Novel**

Novel variants are formatted in bold.

AR, autosomal recessive; dbSNP, single nucleotide polymorphism database; M, maternal; NA, not applicable; P, paternal; XLR, X-linked recessive.

### Special case presentation

#### PCD combined with PNH and PDA (Case 4)

A 2-month-old boy presented with a 6-day history of fever and coughing. At 1 month of age, he was hospitalized for 15 days due to severe pneumonia, respiratory failure, and cardiac failure. Physical examination revealed mild hypotonia and joint hypermobility. Echocardiography identified a PDA measuring 2.1 mm. Cranial MRI showed subependymal nodular gray matter heterotopia involving the body of the left lateral ventricle and the posterior horn of the right lateral ventricle. Given his recurrent pneumonia and the presence of gray matter heterotopia, WES was performed. Genetic analysis identified compound heterozygous variants in the DNAH5 gene, along with a hemizygous variant (c.7760A>C [p.H2587P]) in the FLNA gene—a novel observation inherited from his mother.

The patient was diagnosed PCD combined with PNH and PDA. Over the subsequent 2 years of follow-up, he was hospitalized 4 times due to recurrent pneumonia, and developmental assessments revealed mild delays in milestone achievement.

## Discussion

To the best of our knowledge, this is the largest study to describe the clinical and genetic characteristics of children with PCD in central China. Due to its non-specific symptoms, PCD is often misdiagnosed as chronic bronchitis, chronic pneumonia, or asthma. This misdiagnosis can lead to a delay in receiving the correct diagnosis and management. In our study, the median age of diagnosis was 8.2 years, which is later than the median age of diagnosis reported in European (Kuehni et al., 2010), North American (Welch et al., 2004), and Beijing (Guan et al., 2021), China. Moreover, all PCD patients in this study were diagnosed only after 2018. This diagnostic delay in central China may stem from a lack of appropriate diagnostic facilities, such as transmission electron microscopy (TEM) and genetic testing, which are essential for confirming PCD. Additionally, there is insufficient awareness of the disease among clinicians, which further contributes to the delay.

In this study, the majority of children with PCD were male, with a male-to-female ratio of 2.3:1. This proportion is significantly higher than the reported ratios in North America (0.8:1) (Davis et al., 2015), Europe (1.0:1) (Halbeisen et al., 2019), and other regions of China (1.5:1) (Guan et al., 2021). However, this imbalance in our cohort does not necessarily indicate a higher disease prevalence among males but may be influenced by cultural and socioeconomic factors, particularly in rural families of central China—a major agricultural region—where disparities in healthcare access and prioritization may vary between genders.

Notably, 46.7% of the children had neonatal respiratory distress, which is consistent with the reported rate in Beijing, China (40%), but significantly lower than the reported rate in North America (82%). The lower incidence of neonatal respiratory distress in China compared to North America may be attributed to differences in diagnostic criteria and reporting practices, limited access to specialized neonatal care in developing countries, and genetic and environmental factors.

In this study, except for three children who were under 3 years old, the remaining 12 children all had bronchiectasis, primarily in the middle and lower lobes of the right lung. This finding is consistent with the report by Brown et al. on the locations of bronchiectasis in adults with PCD (Brown et al., 2008). However, bronchiectasis is not specific to PCD. Dettmer et al. reported that the tree-in-bud pattern, mucous plugging, and atelectasis were more common in PCD than in patients with bronchiectasis due to other causes, while emphysematous and fibrotic changes were less common (Dettmer et al., 2018).

Approximately 50% of patients with PCD exhibit left-right laterality abnormalities (Horani and Ferkol, 2021). However, in this study, only 2 cases were complicated by situs inversus, a figure comparable to the 8% reported by Guan et al. in Beijing China (2021) (Guan et al., 2021). This prevalence is significantly lower than the previously reported rates of 55% in North America (2004) (Noone et al., 2004) and 53.3% in England (2019) (Rubbo et al., 2020). While situs inversus in PCD is strongly associated with genetic factors, the precise mechanisms underlying its occurrence remain under investigation (Catana and Apostu, 2017). Variations in prevalence across studies may be influenced more by differences in sample size and regional reporting practices than by true biological variation. Further research is needed to clarify the contributions of genetic factors, ethnicity, and environmental influences to these differences.

In this study, four of the fifteen children underwent gastroenterography. Two cases demonstrated complete visceral translocation, whereas the other two exhibited tortuously elongated duodenum or colon. Existing literature on gastrointestinal phenotypes in children with PCD remains limited. A clinical study by Wee et al., which included 159 cases of PCD, reported a 6% prevalence of gastrointestinal phenotypes, characterized by intestinal malrotation and duodenal atresia (Wee et al., 2022). Therefore, further studies with larger sample sizes are needed to better characterize these morphological variations and their clinical implications.

Interestingly, we identified a unique case (Case 4) of PCD combined with PNH, a rare combination not previously reported in the literature. To date, 10 pathogenic genes have been associated with PNH, with the X-linked filamin A (FLNA) gene being the most common. The majority of patients with FLNA-related PNH are female, as most male patients do not survive beyond the prenatal stage (Loft et al., 2022). However, live-born males with FLNA variants have been reported (Cannaerts et al., 2018). Approximately 90% of PNH patients experience difficult-to-treat seizures. Case 4, a male child, exhibited no history of seizures during the 2-year follow-up period. It has been suggested that some functional FLNA may be produced in male PNH patients who survive infancy (Cannaerts et al., 2018), and that missense variants affecting non-conserved residues may result in only mild alterations to protein folding and binding activity. Case 4 in our study contributes to the expansion of the clinical phenotype spectrum of live-born males with FLNA variants.

In this study, one patient (Case 6) also had an IgG subclass deficiency, which may not be an incidental finding. Bai et al. reported that 11 cases (16.1%) of children with PCD were associated with humoral immunodeficiency, including eight cases of IgG deficiency (Boon et al., 2014). Although the correlation between PCD and IgG deficiency remains unclear, this observation underscores the importance of considering potential immunodeficiency in patients with PCD. For these cases, immunoglobulin therapy may offer significant benefits.

The measurement of nNO is non-invasive, easy to perform, and has been recommended as a screening tool for PCD in Europe and North America. Leigh et al. proposed a threshold of 77 nL/min for nNO, but their study focused on older children and adults who were able to perform breath-holding or whistling techniques during measurement (Leigh et al., 2013). In contrast, younger or uncooperative children can only undergo nNO measurement through tidal breathing. Marthin et al. reported a threshold of 43 nL/min (sensitivity 100%, specificity 100%) for nNO in tidal-breathing children (Marthin and Nielsen, 2013); however, further studies are needed to validate this value. In this study, all measured nNO levels were below 77 nL/min.

In this study, genetic testing was conducted on 14 children, identifying 15 novel variants in five genes through sequencing. Of these, *DNAH5* was the most frequently identified gene, consistent with previously studies (Peng et al., 2022; Emiralioğlu et al., 2020), with a prevalence rate as high as 46.2%, followed by *DNAH11*, *DNAAF2*, *DNAH9*, *DNAAF6*, and *DNAAF3* genes. Recent studies have established a correlation between genotype and phenotype. With the discovery of new genes, the sensitivity of genetic testing has increased. However, 20%–30% of patients diagnosed with PCD based on clinical phenotype and other diagnostic methods have no detectable disease-causing variants (Despotes et al., 2024). Therefore, negative genetic test result cannot exclude PCD. Despite the high cost, genetic testing remains the most important diagnostic method for PCD in our center.

In summary, PCD is underdiagnosed in central China. Additionally, the incidence of neonatal respiratory distress and situs inversus is notably lower compared to Western countries. The most frequently identified gene responsible for PCD was *DNAH5*. Our findings highlight the need for increased awareness and education about PCD among clinicians in China. Establishing specialized diagnostic centers with advanced diagnostic facilities can help reduce the diagnostic delay and improve patient outcomes. Further studies are needed to explore the genetic diversity of PCD in different regions of China and to develop targeted therapies based on the underlying genetic mutations.

## Data Availability

The original contributions presented in the study are publicly available. This data can be found here: National Genomics Data Center (NGDC) - Genome Sequence Archive (Accession number: HRA011710).
